# Supplementary material for: Psychometric properties of the Chinese version physical literacy assessment questionnaire among high school students in Gansu, China
Source: PeerJ. 2025 Apr 9;13:e19093. doi: 10.7717/peerj.19093 (PMC11992972; doi:10.7717/peerj.19093)
Supplement: Supplemental Information 2 [file peerj-13-19093-s002.pdf]

E: The Portuguese Physical Literacy Assessment Questionnaire
Instruction: *Please rate the following statements as they pertain to you with a value of 0,1,2,3 or 4 according to the specified scale.*

Portuguese Physical Literacy Assessment Questionnaire (PPLA-Q)

name: school: gende: Student ID:

For the next question group, you should remember that PA = Physical Activity(ies), and includes every situation that require movement, like your Physical Education classes, sport-based activities (team or individual), rhythmic activities (e.g., dance), exercise (e.g., strength training, jogging), and any activities that you use as a mean of transportation or in your spare time.

In each statement below, select how much it describes you, circling one of the options from 0 to 4 0 = ***Not at all***; 1 = ***Slightly***; 2 =***Moderately***; 3 =***Quite a lot***; 4 = ***Totally***).

How much do these statements describe you?

P1. I am motivated to practice PA	0	1	2	3	4
P2. I practice PA because others tell me I should	0	1	2	3	4
P3. I feel guilty when I do not practice PA	0	1	2	3	4
P4. I feel bad about myself when I do not practice PA	0	1	2	3	4
P5. I feel pressured by others to practice PA	0	1	2	3	4
P6. I practice PA because I feel others would be unhappy if I did not	0	1	2	3	4
P7. I practice PA because it is fun	0	1	2	3	4
P8. I feel good when I practice PA	0	1	2	3	4
P9. I consider PA a part of me	0	1	2	3	4
P10. I value the benefits of PA	0	1	2	3	4
P11. I see PA as a fundamental part of who I am	0	1	2	3	4
P12. I enjoy practicing PA	0	1	2	3	4
P13. I feel confident to practice PA	0	1	2	3	4

In Physical Activity contexts:

P14. I am confident in my abilities	0	1	2	3	4
P15. I can participate with success	0	1	2	3	4
P16. I consider myself competent	0	1	2	3	4
P17. I trust my skills	0	1	2	3	4
P18. I feel good about the way I am able to participate	0	1	2	3	4
P19. I can participate in PA that I consider challenging	0	1	2	3	4
P20. I know how to become more confident in myself	0	1	2	3	4
P21. I feel competent even when I am criticized	0	1	2	3	4

P22. I believe in myself even when I lose	0	1	2	3	4
P23. I can manage my emotions	0	1	2	3	4
P24. I can recognize other's emotions	0	1	2	3	4
P25. I can recognize my emotions	0	1	2	3	4

In Physical Activity contexts:

P26. I am sensitive to the feelings of others	0	1	2	3	4
P27. I understand what others feel	0	1	2	3	4
P28. I can identify what I feel	0	1	2	3	4
P29. I can anticipate what I will feel	0	1	2	3	4
P30. I can deal with difficulties rationally	0	1	2	3	4
P31. I can manage my emotions when necessary	0	1	2	3	4
P32. I have a good control of my emotions	0	1	2	3	4
P33. I can manage my effort	0	1	2	3	4
P34. I know when I am tired	0	1	2	3	4
P35. I can recognize changes in my breathing	0	1	2	3	4
P36. I can recognize changes in my heart rate	0	1	2	3	4
P37. I recognize my physical limits	0	1	2	3	4
P38. I can recognize the effect that different intensities have in me	0	1	2	3	4
P39. I use strategies to manage my effort	0	1	2	3	4
P40. I can anticipate when I will be fatigued	0	1	2	3	4
